# The sense of body ownership relaxes temporal constraints for multisensory integration

**DOI:** 10.1038/srep30628

**Published:** 2016-08-03

**Authors:** Antonella Maselli, Konstantina Kilteni, Joan López-Moliner, Mel Slater

**Affiliations:** 1 Event Lab, Department of Personality, Evaluation and Psychological Treatment, Universitat de Barcelona, Barcelona, 08053, Spain; 2 Laboratory of Neuromotor Physiology, Santa Lucia Foundation, Rome, 00179, Italy; 3 Vision & Control of Action Lab, Department of Psicologia Bàsica, Universitat de Barcelona, 08035, Catalonia, Spain; 4 Institut de Neurociències, Universitat de Barcelona, Spain; 5 Institució Catalana de Recerca i Estudis Avançats (ICREA), Barcelona, 08010, Spain; 6 Department of Computer Science, University College London, London, UK

## Abstract

Experimental work on body ownership illusions showed how simple multisensory manipulation can generate the illusory experience of an artificial limb as being part of the own-body. This work highlighted how own-body perception relies on a plastic brain representation emerging from multisensory integration. The flexibility of this representation is reflected in the short-term modulations of physiological states and perceptual processing observed during these illusions. Here, we explore the impact of ownership illusions on the temporal dimension of multisensory integration. We show that, during the illusion, the temporal window for integrating touch on the physical body with touch seen on a virtual body representation, increases with respect to integration with visual events seen close but separated from the virtual body. We show that this effect is mediated by the ownership illusion. Crucially, the temporal window for visuotactile integration was positively correlated with participants’ scores rating the illusory experience of owning the virtual body and touching the object seen in contact with it. Our results corroborate the recently proposed causal inference mechanism for illusory body ownership. As a novelty, they show that the ensuing illusory causal binding between stimuli from the real and fake body relaxes constraints for the integration of bodily signals.

During body ownership illusions (BOIs) healthy adults experience artificial limbs or bodies as part of their own body representation. BOIs are thought to rely on the integration of synchronous but independent perceptual stimuli, for example visual stimuli seen on the dummy hand and tactile ones felt on the real hand in the case of the Rubber Hand Illusion (RHI)^1^. Similar multisensory manipulations have been shown effective to induce the illusion of ownership over supernumerary limbs^2^,^3^,^4^ and artificial bodies^5^,^6^, demonstrating how our own-body perception relies on a plastic brain representation emerging from multisensory integration^7^,^8^,^9^,^10^.

Evidence for the flexibility of this brain representation comes not only from vivid subjective reports about illusory ownership experienced over artificial limbs and full bodies, whose appearance can importantly deviate from the one of the real counterpart (e.g. in size^11^,^12^,^13^, skin color^14^,^15^, age^16^ and realism^17^,^18^). This flexibility is also robustly supported by a number of short-term modulations of behavior and physiological states. As representative examples, it has been reported that during a RHI the temperature of the “substituted” hand drops^19^ together with its tactile sensitivity^19^,^20^,^21^, and that histamine reactivity to noxious stimuli increases^22^. On the behavioral side, it was shown, for example, how experiencing ownership over a dark-skin body can reduce implicit racial-bias^15^,^23^, or how ownership over a child body can affect size perception and induce implicit attitude changes^16^. These are just some examples of the profound impact on self-perception and behavior that can occur during body ownership illusions, over time scales of few tens of seconds.

Multisensory integration has been at the core of research into body ownership illusions since their first reports^1^,^7^ and is indeed regarded as the basic causal mechanism for their emergence^9^,^24^,^25^. However, the converse has not been previously studied, which is concerned with how BOIs modulate the processing and merging of cross-modal stimuli. On the other hand, extensive research in multisensory perception shed light over the intrinsic flexibility of multisensory integration processing, which allows adapting efficiently to the continuously changing environment. In this study we face this gap and explore potential effects of ownership illusions on multisensory processing.

Integrating the different multimodal signals streaming from an object or an event is essential for having a coherent and meaningful perceptual experience, but this is not a straightforward task for the central nervous system (CNS). In fact, due to differences in transduction and transmission characteristic times, the temporal lag for a signal to trigger a response in the corresponding primary sensory cortex can significantly vary across modalities. For example, due to the long transduction time of photoreceptors^26^ (longer with respect to that of tactile receptors), a truly synchronous visuotactile event typically triggers activity in SI in advance with respect to activity triggered in V1. Furthermore, the relative delay can change importantly according to the lighting conditions and the ambient temperatures (which affects transduction)^27^, and also depending on the body part that is touched (which affects transmission time of tactile stimuli)^28^. Therefore, in order to assure a coherent perception of the environment, the CNS has to flexibly compensate for such differences by modulating the temporal window on which multisensory integration is effective^29^,^30^,^31^. One of the factors that efficiently modulates the temporal window for multisensory integration is “causal binding”, also referred to as “unity assumption”. Having implicit knowledge of the existence of a common origin for two sensory signals was indeed shown to facilitate integration. In the case of audiovisual speech perception, larger temporal lags are indeed tolerated (perceived as synchronous) under the assumption of a common cause for the visual and auditory signals^32^.

In the light of the results discussed, we argue that body ownership illusions could act as a “causal binding” factor for stimuli seen on the fake body and independent somatosensory signals, and therefore that BOIs could modulate multisensory processing. As a further support to this proposal, a number of studies showed that, during BOIs, visual threats to the artificial body trigger enhanced neurophysiological correlates of anxiety^33^,^34^, thus revealing how visual stimuli on the fake body are processed as if seen on the own physical body. In the present work, we focus on the temporal constraints for visuotactile integration. We specifically hypothesized that, during the illusion, there is an expansion of the temporal window within which visual (on the fake body) and tactile (on the physical body) stimuli are perceived as simultaneous.

To test this hypothesis we performed two experiments. In Experiment 1, we assessed whether the assumption of a common origin – for a tactile stimulus and a correspondent visual cue on the fake body – could expand the temporal window of integration in the visuotactile domain, similarly to what has been found for the audiovisual domain^32^,^35^. In Experiment 2, we tested whether this “causal binding” effect emerges as a consequence of the body ownership illusion itself.

We relied on a temporal order judgment (TOJ) task as an established procedure to estimate the temporal window of integration. In TOJ tasks, pairs of target stimuli with varying temporal separations (typically referred to as Stimulus Onset Asynchrony – SOA) are presented to participants that have to judge which stimulus came first. The analysis of how responses vary across the tested range of SOAs, allows estimating two main quantities of interest: the Point of Subjective Simultaneity (PSS) – a measure of the average time one stimulus has to precede the other in order for the two to be perceived as simultaneous^36^,^37^– and the Just Noticeable Difference (JND) – a measure of the temporal window in which the two stimuli are perceived as simultaneous^38^,^39^,^40^. In particular, for a multimodal TOJ task, the JND can be regarded as a proxy for the temporal window of multisensory integration^32^,^41^,^42^, and as such it is the quantity of interest for the current study.

We implemented a visuotactile TOJ task that participants had to perform while wearing a head-tracked head-mounted display (HMD) streaming a digital 3D replica of the experimental room. When looking down towards their real occluded body, participants could see either a virtual body or two wooden sticks, depending on the experimental condition. Participants performed the TOJ task while resting their arms on a Table (Fig. 1A). The tactile stimulus was a 50 ms vibration delivered to the right index fingertip. The visual stimulus was a rotation (50 ms duration) of a virtual geared-wheel (Supplementary Video S1). For further details on the TOJ task implementation and on methods for estimating the JNDs, we refer the reader to Materials and Methods.

In Experiment 1, we explicitly manipulated the causal relationship between the visual and tactile stimuli. Participants had a first person perspective over a gender-matched virtual body that was spatially coincident with their real body. This configuration is known to be sufficient for inducing an ownership illusion through congruent visuo-proprioceptive cues^6^,^43^. Participants performed the TOJ task in two conditions: the geared-wheel was seen either in contact with the index finger of the virtual body (*Touch*) (Fig. 1C) or separated from it by 6 mm (*No-Touch*) (Fig. 1D, Supplementary Video S1).

In Experiment 2, we tested whether this illusory “causal binding” is mediated by the ownership illusion. Participants performed the TOJ task in two conditions with different manipulations of illusory body ownership. One condition (*Body*) was the same as the *Touch* one in Experiment 1 (Fig. 1C). In the other (*Sticks*), the sense of ownership was inhibited by displaying two wooden sticks placed on the Table and spatially coincident with the arms of the participants during the TOJ task, with the tip of the right stick being in touch with the geared-wheel (Fig. 1E, Supplementary Video S1). This manipulation of the shape of the virtual body was used since successful induction of a BOI requires that the fake body or body part should have a humanoid shape^5^,^44^.

A six-item questionnaire, presented at the end of each experimental session, was administered to assess the subjective illusory experience (Table 1). Each item was scored on a −3 to +3 Likert scale, according to the level of agreement with the statement.

## Results

In Experiment 1 we expected to find higher JND in the *Touch* condition, because seeing the virtual finger touching the moving geared-wheel during an ownership illusion should provide hints for a common origin of the two target stimuli. This was indeed the case for 10 out of 14 subjects, with an average JND difference across conditions of 46 ms (Fig. 2A,B). Data from all 14 participants were analyzed using a Generalized Linear Mixed Model (GLMM). The GLMM analysis revealed a significant difference across conditions in the slopes of the fitted psychometric curves (p < 0.0001). Corresponding bootstrap estimates of the JNDs were 155 ms (95% CI = 142 to168 ms) and 127 ms (95% CI = 117 to 136 ms) in the *Touch* and *No-Touch* conditions respectively (Fig. 2C).

Results from the subjective scores to the six-item questionnaire are summarized in Table 2. Participants reported a strong illusion of virtual body ownership (median scores of 2) in both conditions (p > 0.48). The feeling that the wheel was directly touching their finger was positively reported only in the *Touch* condition (p = 0.002; median scores of 1.5 for *Touch* and −3 for *No-Touch*). Consistently, the feeling of the virtual wheel being the origin of the vibrotactile TOJ cues was higher in the *Touch* condition (median scores of 1 for *Touch* and −0.5 for *No-Touch*), although the difference between conditions did not reach the significance level (p = 0.06). These results support our hypothesis that the illusory experience of touching the virtual wheel acts as a “causal binding” factor, expanding the temporal window for visuotactile integration.

In Experiment 2 we explicitly manipulated illusory body ownership to test the hypothesis that the illusion itself mediates the “causal binding” effect observed in Experiment 1. Results from the subjective scores to the questionnaire items are summarized in Table 2. Participants experienced a strong sense of ownership only in the *Body* condition (median scores of 2 for *Body* and −2 for *Stick*; p = 0.005). Analogously, the reported sensation of touching the geared-wheel (*touch* item) was high only for the *Body* condition (median scores of 1.5 for *Body* and −2 for *Stick*; p = 0.01). Contrary to our expectation, this was not reflected in a significant difference for the reported sensation that the geared-wheel was the cause of the vibrotactile stimuli during the TOJ (*cause* item: p = 0.12).

Importantly, for 11 of the 14 participants the JND was larger in the *Body* condition, with an average JND difference across conditions of 16 ms (Fig. 3A). Analysis of the 14 subjects performance using GLMM showed a significant difference across conditions for the fitted slope of the psychometric curves (p = 0.011), with bootstrap estimates of the JNDs being 117 ms (95% CI = 95–131 ms) and 105 (95% CI = 83–118 ms) in the *Body* and *Sticks* conditions respectively (Fig. 3B). Crucially, the JND was positively correlated with subjective scores rating the sense of ownership (*r*_*S*_ = 0.41, p = 0.033), the illusory sensation of touching the geared-wheel (*r*_*S*_ = 0.46, p = 0.014), and of the geared-wheel being the cause of the vibrotactile target cues (*r*_*S*_ = 0.46, p = 0.014) (Fig. 3C). This latter result may seem at odd with the unexpected lack of significant difference across conditions in the *cause* item scores. In fact, according to the rationale of our hypothesis, the illusion of ownership (triggered by the view of the spatially overlapping virtual body and reinforced by synchronous visuomotor stimuli) would generate the illusion of touching the virtual geared-wheel. This illusory touch would then trigger the illusory experience of the geared-wheel being the origin of the vibrotactile stimuli and in turns would relax temporal constraints for visuotactile integration (measured in terms of JNDs). In order to test this chain of predictions more thoroughly, we further computed Spearman correlations across questionnaire items. As expected, significant positive correlations were found between scores to *move* and *ownership* items (*r*_*S*_ = 0.69, p < 0.0001), *ownership* and *touch* items (*r*_*S*_ = 0.58, p = 0.0015) and *touch* and *cause* items (*r*_*S*_ = 0.57, p = 0.0019). The correlation between scores to *ownership* and *cause* instead was not significant, but just a trend (*r*_*S*_ = 0.29, p = 0.14). All results from Spearman correlations analysis are summarized in Figure S1. It should be noticed that, even if on average participants did not report ownership towards the wooden sticks, few subjects (three out of thirteen) reported a positive sense of ownership also in the *Sticks* condition. Interestingly, for these cases the corresponding illusory touch and causal association of the experienced touch with the seen wheel rotation was positive (Figure S2). This could explain why the experimental manipulation did not significantly affect scores to the *cause* item, while the large variance of the cause item scores in both Stick and Body conditions could explain why the positive trend found in the correlation between *ownership* and *cause* items did not reach significance (Table 2, Figure S2).

Results from Experiment 2 thus support our hypothesis, indicating that the effect of “causal binding” on the temporal window of visuotactile integration revealed in Experiment 1 is selectively mediated by the sense of ownership experienced toward the virtual body.

Taken together results from Experiment 1 and 2 highlight the reciprocal connection between multisensory integration and body ownership: multisensory integration builds the body ownership illusion, while the illusion, once induced, modulates subsequent multisensory processing.

## Discussion

Spatiotemporal correlations of concurrent stimuli from different sensory channels and the motor system provide an essential contribution to self-perception and self-recognition^45^,^46^,^47^. Experimental protocols for ownership illusions rely on this principle: the spatiotemporal congruence of cross-modal bodily signals triggers the integration of truly independent stimuli, giving rise to the illusory experience that an external object is part of the own-body^6^,^9^,^25^,^48^.

Here we provide novel experimental evidence that illusory ownership modulates multisensory integration. The results of the two experiments combined show that during the illusion the temporal window for visuotactile integration of body related cues expands. In the first experiment, we induced an ownership illusion over a virtual body so to be able to dissociate visual and tactile bodily signals: participants could see a rotating geared-wheel in touch or not with the virtual finger while receiving tactile stimuli on the real fingertip. When the finger was seen in touch with the moving object, the estimated temporal window for visuotactile integration was wider. This showed that when pairs of visuotactile bodily stimuli are attributed to the same cause, the temporal constraints for their integration get relaxed.

An alternative interpretation would be that visuotactile integration is inhibited in the *No-Touch* condition because of the spatial offset between visual and the tactile stimuli. While in the *Touch* condition the visual and tactile stimuli were aligned in space, in the *No-Touch* condition a small offset (6 mm) was introduced between the two, as the virtual finger was slightly displaced (with respect to the real one) so to be seen separated from the virtual wheel. Previous studies have shown that the perceived distance between target-stimuli can significantly affect TOJ performances, with better performances found for more distant stimuli^39^,^40^. Notwithstanding, in our case the change in distance between the visual and tactile stimuli across conditions was extremely small, and of the same order of magnitude of the intrinsic precision of the hand position sense^49^. Therefore the introduced offset should not significantly affect the perceived location of the tactile stimuli, remapped in external space towards the location of the slightly displaced virtual finger. It is worth noticing that previous studies reported changes in JND, as estimated from TOJ performances, of the order of 10 ms for an actual change in the target-stimuli distance of about 1 m^39^, while here we found a change in JND almost three times larger for an actual change in distance of just 6 mm. We can therefore conclude that the change in JND we found is not an effect of different displacements between target stimuli, but is instead due to the illusory causal binding elicited in the *Touch* condition and absent in the *No-Touch* condition.

In the second experiment, we manipulated illusory body ownership to show that the effect of causal binding observed in Experiment 1 is selectively modulated by the sense of ownership. We found that, if the sense of ownership is inhibited (by showing a wooden stick instead of the virtual hand touching the geared-wheel) the temporal window for visuotactile integration is indeed smaller with respect to the case in which the body ownership illusion was strong.

All together, results from our two experiments suggest that the relaxation of temporal constraints for multisensory integration observed in body ownership illusions is driven by a causal mechanism that binds together visual stimuli on the fake body and tactile stimuli on the physical body.

Interestingly, our results could explain why participants undergoing an intense ownership illusion fail to notice asynchronies in visuotactile stimulation that are otherwise (in the absence of the illusion) detected^6^. Furthermore, the present results provide robust experimental support to previous proposals suggesting that the illusion can be sustained despite exposure to asynchronous visuotactile stimulation^6^,^10^.

Our results fit well within the framework provided by Bayesian causal inference models for illusory ownership^10^,^50^. According to such models, the illusion arises when the brain associates a higher than chance probability to the existence of a single cause (the own-body) for all the incoming sensory input: the visual from the fake body and the somatosensory/motor from the physical body. Importantly, the causal inference approach predicts that, under the assumption of a common cause, the integration of cross-modal sensory stimuli is facilitated^51^,^52^,^53^. This prediction is indeed corroborated in the present study that showed facilitation in terms of an expanded temporal window for visuotactile integration.

The influence of causal binding on temporal aspects of multisensory perception has been previously reported. In the audiovisual domain, similar expansions of the temporal window of integration have been found, but only for functionally relevant stimuli such as speech^32^,^35^. Similarly, intentional binding – a form of causal binding manipulated through voluntary motor actions – has been shown to anticipate the conscious perception of auditory stimuli^54^ and to suppress color-motion asynchronies^55^, otherwise observed in the perception of truly simultaneous changes in the color and position of an object^56^. These results highlight the predictive nature of causal binding effects: predictions are indeed an intrinsic component of both voluntary motor control^57^,^58^ and speech perception^59^. In line with these findings, our results suggest that the sense of body ownership involves predictive cause-effect mechanisms that shape the processing of bodily signals during body-environment interactions. Notably, we show that such predictive mechanisms, which have the functional role of preserving and guiding the physical body through the environment^60^, are operating likewise during body ownership illusions.

The modulation of visuotactile processing reported in this study relies probably on the same mechanisms for simultaneity constancy, which allows correct perception of truly synchronous cross-modal stimuli despite intrinsic differences in their processing latencies^28^,^29^. Mechanisms suggested for such temporal compensation, including sliding temporal binding windows^61^ or temporal ventriloquisms^62^, provide phenomenological descriptions but hardly insights into their neurophysiological basis. Although indirectly, our results provide support for a top-down modulation of multimodal neurons processing bodily signals^7^,^63^, and strongly indicate that such modulation can happen on the short time-scale of few tens of seconds.

In conclusion, our results provide experimental evidence that body ownership illusions affect multisensory integration. We have shown that, by establishing a causal link between the fake and physical bodies, the sense of body ownership enhances the temporal flexibility of visuotactile integration. This adds an important contribution to previous results on the sense of body ownership and ownership illusions. It was known that short periods of conflictive multisensory stimulations can induce dramatic illusory changes in our own-body representation, with important consequences at physiological^19^,^22^, psychological^64^ and behavioral^15^,^16^ levels. Here we show that changes occur also at the level of basic processing of multisensory information, which is relevant for the interaction of the body with the environment. Furthermore, these results demonstrate how the sense of body ownership guarantees a flexibility in visuotactile integration that goes beyond what is required in our normal daily experience where visual and tactile stimuli from the body are locked in space and time.

## Materials and Methods

### Participants

Fourteen healthy subjects (8 female; mean age ± SD: 20.7 ± 2 years) took part in Experiment 1 and another fourteen in Experiment 2 (8 female; mean age ± SD: 21.6 ± 4.6). No participant had a history of neurological disease and all had normal or corrected-to-normal vision. They signed an informed consent form and received 10 euros as compensation. The experimental protocol was approved by the “Ethical Committee for research” of the University of Barcelona, in line with the institutional ethics and national standards for the protection of human participants.

### Experimental Setup

**Figure 1:**
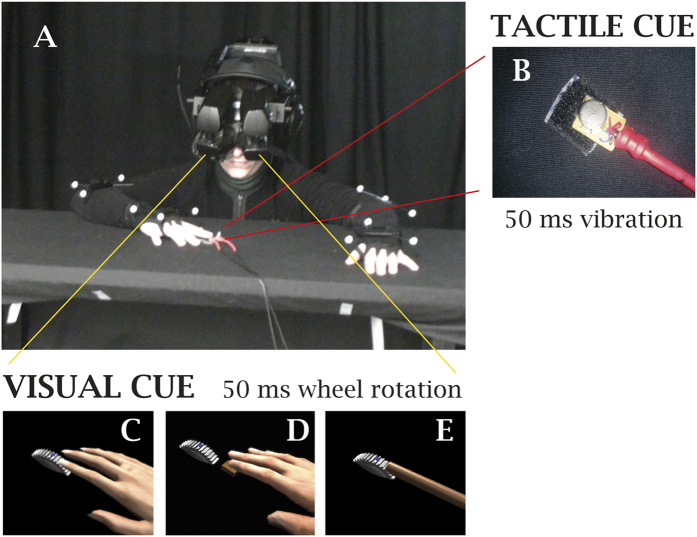
Experimental setup. **(A)** Participants performed a visuotactile Temporal Order Judgment (TOJ) task, while wearing head-mounted display. (**B)** Piezoelectric motor used to deliver vibrotactile stimuli. The visual stimulus was a 50 ms rotation of a virtual geared-wheel: the geared-wheel was seen (**C**) in contact with the virtual finger (Exp. 1: *Touch* condition; Exp. 2: *Body* condition), (**D**) separated from the virtual finger (Exp. 1: *No-Touch* condition) or (**E**) touching a wooden stick (Exp. 2: *Stick* condition). The 3D graphics elements were designed with Autodesk^®^ 3ds Max^®^ and controlled through the Unity^®^ software platform.

Participants sat in front of table where a coin-vibrator was placed along the participant’s sagittal plane (Fig. 1A). A wide field-of-view, stereo head-tracked, head-mounted display (HMD) was used to stream in 3D a virtual reproduction of the experimental room. By moving their head, participants could explore the environment. According to the experimental condition, when looking down towards their own body participants saw either a gender-matched virtual body from a first-person-perspective and spatially coincident with their physical body, or two virtual sticks placed on the table. Participants’ head and arms movements were tracked via a combination of infrared cameras and inertial devices. Vibrotactile stimulations were delivered via the coin-vibrator (Fig. 1B) controlled through an Arduino board. Details on the devices specifics, the VR project implementation and the setup validation are available in the Supplemental Information and in Supplementary Table S1.

### Temporal Order Judgment (TOJ) Task

The TOJ task consisted in a forced choice discrimination of the temporal order of two target cues. The tactile one was a 50 ms long vibrotactile stimulus (single burst) delivered on the fingertip of the right index by a piezo-electric motor (0.78 cm^2^, 1200 ± 300 r.p.m.). The visual cue consisted in a 50 ms long rotation of a virtual geared-wheel object about its axis (Supplementary Video S1). A complete session included 200 trials spanning a SOA range of [−600, 600], sampled at {±600, ±300, ±200, ±80} ms. Each trial was presented 1200 ms after receiving the participant’s response to the previous trial. The visual and tactile stimuli were spatially aligned in external space in all conditions but the *No-Touch* one in Experiment 1. In the latter case, the rotating wheel was seen slightly displaced from the virtual finger (6 mm), so that a small offset was introduced between the visual and tactile TOJ target-cues. This was implemented by slightly displacing the right virtual arm, during the TOJ, so that the tip of the virtual index was 6 mm away from the tip of the real index in the direction of the interphalangeal joint. During the task participants were instructed to keep their arms and head still, and fix their gaze on a blue dot displayed on the geared-wheel (Fig. 1C–E; Supplementary Video S1). They used two pedals to provide responses.

### Procedure

Participants wore the HMD and headphones streaming white noise. First, they familiarized with the environment and performed a TOJ training session (25 trials). Next, they underwent two experimental sessions, counterbalanced across participants. The 200 TOJ trials were presented in blocks of 25 trials, alternated with breaks in which participants were asked to mark with their hands the position of a cross appearing on the table at different positions (Supplementary Video S2). In all conditions, but *Stick* in Experiment 2, the virtual body moved along with the participants tracked-movements. These breaks were included to make participants move their head and arms, so to relax tension and to keep high the sense of body ownership through congruent visuomotor correlations^65^. Each session had an average duration of 18 minutes. After its completion, participants filled a 5-item questionnaire customized to assess different aspects of the subjective illusory experience (Table 1).

### Analysis

Questionnaire scores across conditions were compared with the Matched-pairs Wilcoxon tests, and the associated effect size was quantified in terms of “probability of superiority” of dependent measures (PS_dep_)^66^.

TOJ responses, from each subject and condition, were converted into probabilities of “touch-first” response, *P*(*Y*_*j*_), at each SOA point tested. These values were next fitted with a psychometric curve of the form:


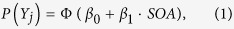


where 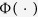 denotes the probit function. The temporal window of integration was then estimated in terms of the Just Noticeable Difference (JND), by definition proportional to the inverse of the slope, *β*_1_, and corresponding to the 75% threshold in the probability distribution^67^.

**Figure 2:**
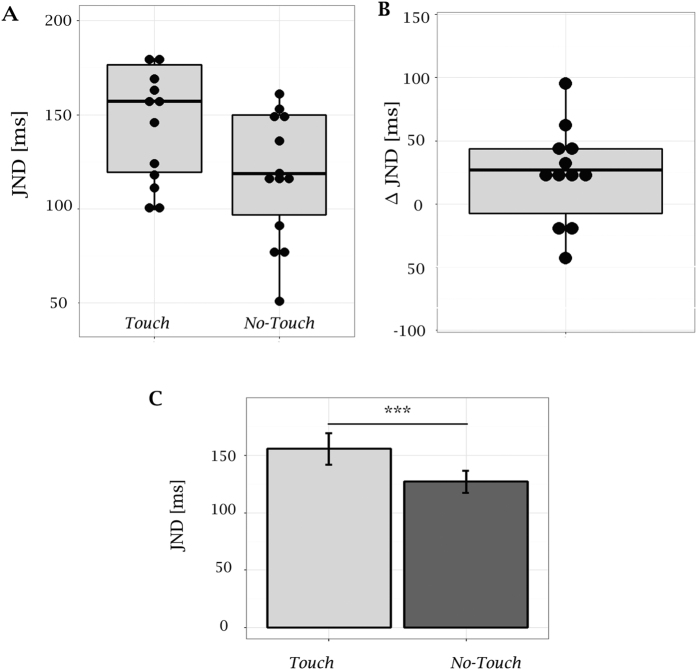
Results from Experiment 1. **(A)** Boxplot showing the distribution of JND estimates from individual fits in the two experimental conditions; *JND* estimates from single subjects are overplotted as scatter points. **(B)** Boxplot showing the distribution of JND differences in the two conditions (*ΔJND* = *JND*_*Touch*_* − JND*_*No-Touch*_), estimated from individual fits; *ΔJND* for single subjects are overplotted as scatter points. For 10 out 14 participants the JND was higher in the *Touch* condition. The mean value of individual *ΔJND* was 46.6 ms. **(C)** JNDs estimates (n = 14) from Genelized Linear Mixed Model (Bootstrap method) were equal to 127 ms and 155 ms in the *No-Touch* and *Touch* conditions respectively. Vertical bars represent the 95% CI estimated with the bootstrap method^67^. GLMM analysis revealed a significant difference in across conditions (p < 0.0001).

**Figure 3:**
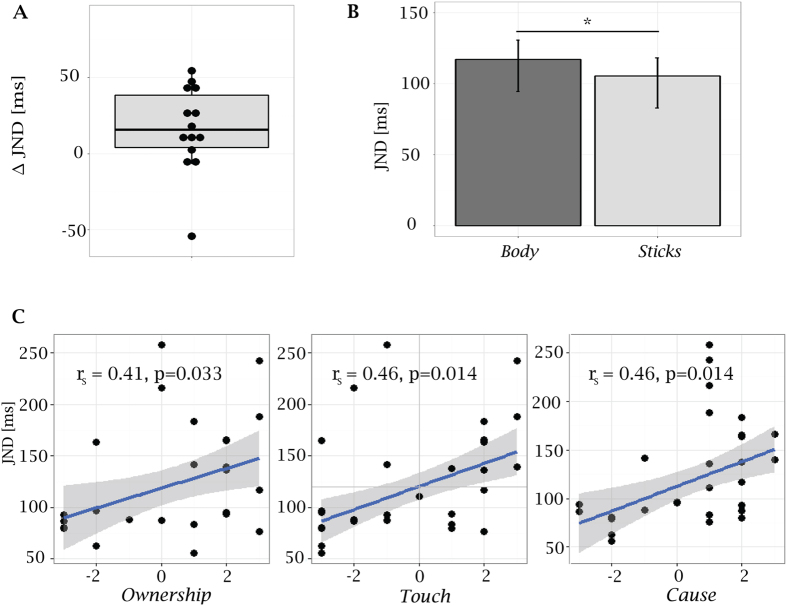
Results from Experiment 2. **(A)** Boxplot showing the distribution of JND difference in the two conditions (*ΔJN*_*D*_ = *JND*_*Body*_ *−* *JND*_*Sticks*_), estimated from individual fits; *ΔJND* for single subjects are overplotted as scatter points. For 11 out of 14 participants the JND was higher in the *Body* condition. The mean value of individual *ΔJND* was 16.3 ms. **(B)** JNDs estimates (n = 14) were equal to 127 ms and 155 ms in the *Stick* and *Body* conditions respectively. Vertical bars represent the 95% CI estimated with the bootstrap method^67^. GLMM analysis revealed a significant difference across conditions (p = 0.011). **(C)** JND estimates from individual fits are plotted as a function of subjective scores given to the “*Ownership*” (left panel), “*Touch*” (central panel), and “*Cause*” (right panel) questionnaire items (full statements listed in Table 1), together with the robust linear fits and associated 95% CIs. Spearman correlation analysis revealed significant positive correlations for the three cases.

These fits provided the individual JND estimates adopted in the correlation analysis and used to generate the boxplots in Figs 2A,B and 3A. Correlations among questionnaire scores and individual JND estimates were assessed in terms of Spearman rank coefficients.

At the group level, TOJ data were analyzed with a Generalized Linear Mixed Model (GLMM) that extends model (1) to include fixed effects associated to the experimental manipulation, and random effects associated to the variability within and between subjects^68^. The advantage of GLMMs with respect to group analysis based on parameters extraction from single subject fits, is that they take into account both inter and intra subject variability and have a higher statistical power^67^. Estimates of the JNDs and the associated 95% CIs were computed with the bootstrap method^67^.

Statistical analysis was performed in R. The GLMM analysis was performed using the glmer function from the lme4 R package^69^.

## Additional Information

**How to cite this article**: Maselli, A. *et al*. The sense of body ownership relaxes temporal constraints for multisensory integration. *Sci. Rep*. **6**, 30628; doi: 10.1038/srep30628 (2016).

## Supplementary Material

Supplementary Information

Supplementary Movie S1

Supplementary Movie S2

## Figures and Tables

**Table 1:** Questionnaire items.

Tag	Questionnaire Item
*Ownership*	During the temporal order judgment task I felt as if the *virtual hand* (stick) I was looking at was my own hand
*Touch*	During the temporal order judgment task I felt as if my right index finger was touching the virtual wheel
*Cause*	During the temporal order judgment task I felt as if the rotation of the virtual wheel produced the tactile stimuli on my finger
*Move*	I felt as if the *virtual arms* (sticks) were following the movements of my own arms
*Ctrl*	I felt as if I had two right hands

The Table lists the six items presented to participants at the end of each experimental session. The items were presented in a randomized order across participants and experimental conditions. Participants had to indicate their level of agreement with each of the statement, on a Likert scale from −3 to 3. The text in parentheses was used to replace the italic text in the *Stick* condition in Experiment 2.

**Table 2:** Questionnaire Results.

*Experiment 1*					
Item	*Touch*	*No-Touch*	*Touch – No-Touch*	p-value	PS_dep_
*Ownership*	2 (1)	2 (1.5)	0 (0.75)	0.48	0.53
*Touch*	1.5 (4.5)	−3 (1)	2.5 (3.75)	0.002	0.96
*Cause*	1 (2)	−0.5 (4.5)	1 (3)	0.06	0.75
*Move*	3 (1)	3 (1)	0 (0.75)	0.78	0.46
	−3 (1.75)	−3 (0)	0 (0.75)	0.10	0.64
***Experiment 2***					
**Item**	***Body***	***Stick***	***Body – Stick***	**p-value**	**PS_dep_**
*Ownership*	2 (1)	−2 (3)	3 (4)	0.005	0.84
*Touch*	1.5 (3)	−2 (3.75)	1.5 (3)	0.01	0.79
*Cause*	1 (1.75)	1 (3.5)	0 (1.75)	0.12	0.61
*Move*	3 (1)	−3 (1.75)	5 (2)	0.001	0.06
*Ctrl*	−3 (0)	−3 (0)	0 (0)	0.58	0.50

The Table lists the median scores assigned to each questionnaire item across participants, together with the associated inter quartile range (IQR) in parentheses, for each condition of the two experiments (columns 2 and 3). Column 3 gives the median (IQR) values of the differences among scores given in the two experimental conditions by each subjects. Columns 4 and 5 give the p-values from Matched-Paired Wilcoxon test and the associated effect size in terms of probability of superiority for dependent measures (PS_dep_).
