# Supplementary material for: The sense of body ownership relaxes temporal constraints for multisensory integration
Source: Sci Rep. 2016 Aug 3;6:30628. doi: 10.1038/srep30628 (PMC4971486; doi:10.1038/srep30628)
Supplement: Supplementary Information [file srep30628-s1.pdf]

Supplementary Information

The sense of body ownership relaxes temporal constraints for multisensory integration

Antonella Maselli[1,2*], Konstantina Kilteni[1], Joan López-Moliner[3,4], Mel Slater[1,4,5,6]

Author Affiliations

[1] Event Lab, Department of Personality, Evaluation and Psychological Treatment, Universitat de Barcelona, Barcelona, 08053, Spain

[2] Laboratory of Neuromotor Physiology, Santa Lucia Foundation, Rome, 00179, Italy

[3] Vision & Control of Action Lab , Department of Psicologia Bàsica, Universitat de Barcelona, 08035, Catalonia, Spain

[4] Institut de Neurociències, Universitat de Barcelona, Spain

[5] Institució Catalana de Recerca i Estudis Avançats (ICREA), Barcelona, 08010, Spain

[6] Department of Computer Science, University College London, London, UK.

Supplementary Information

Material and experimental setup

The technical equipment and software tools used in the experiments are listed in Table S1. The virtual scenario was programmed with the Unity® software platform, using 3D graphics elements designed with Autodesk® 3ds Max®. The head mounted display has a 102°H×64°V total field-of-view, 1280×1024 resolution per eye and 60 Hz frame rate. The coin-vibrator used to deliver vibrotactile stimuli has the following specifics: 78 cm^2, 3V, 100mA, 50Ω, 1200 ± 300 r.p.m. The table was high enough (94 cm) to avoid participants having to bend their neck down during the temporal order judgment (TOJ) task (Figure 1A). The coin-vibrator was placed along the participant's sagittal plane at 24 cm from the table edge.

System Calibration

An oscilloscope (HAMEG Instruments) was used to measure the relative latencies of the visual and tactile target cues in the TOJ task, and to verify their effective duration (50 ms). When triggered at the same timeframe from the Unity® software script, the presentation of the visual cue on the HMD had a 30 ms time lag with respect to the onset of the vibration. This delay was compensated ad hoc in the Unity® script, so that the coded stimulus onset asynchrony (SOA) was precisely controlled.

Temporal Order Judgment (TOJ) task: SOA sampling

The TOJ task consisted of 200 trials spanning a SOA range of [-600, 600] ms and sampled at {±600, ±300, ±200, ±80} ms, with associated sampling frequency of {10, 20, 30, 40}. The SOA order was arranged in 10 cycles of 20 trials each; each cycle included all the sampled SOA values, one to four times according to their corresponding sampling frequency. Trials within each cycle were presented in a random order, independently for each subject and condition.

Table S1. Technical equipment and software tools

Purpose	Name	Name of manufacturer	Web site of product
HMD	SX111 Head-Mounted Display	NVIS	http://nvisinc.com/product.php?id=48
Vibrators	Mechanical coin type vibrators	Shenzhen Linglong Electronics Co	http://linlongelectronics.company.weiku.com/item/Shenzhen-Linglong-Electronics-Co-Ltd-15988395.html
Microcontroller for vibrators	Arduino Mega	Arduino	https://www.arduino.cc/en/Main/ArduinoBoardMega2560
3D modeling	Autodesk® 3ds Max®	Autodesk, Inc	http://www.autodesk.com/products/3ds-max/overview
VR platform	Unity® software	Unity Technologies	http://unity3d.com/

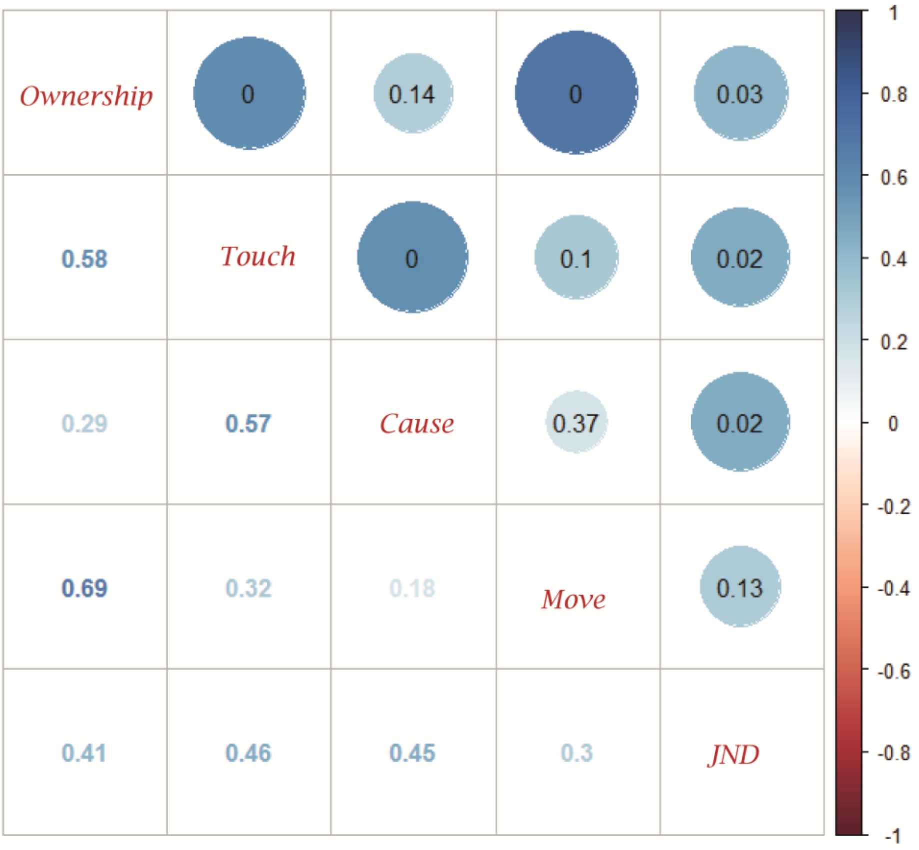

Figure S1. Experiment 2 correlation matrix. The figure reports in a matrix form the correlation coefficients (lower left triangle) and the corresponding p-values (upper-right triangle) among paired combinations of scores to questionnaire items and JND estimates. The color and size of the circles in the upper-right triangle scale with the corresponding correlation coefficient.

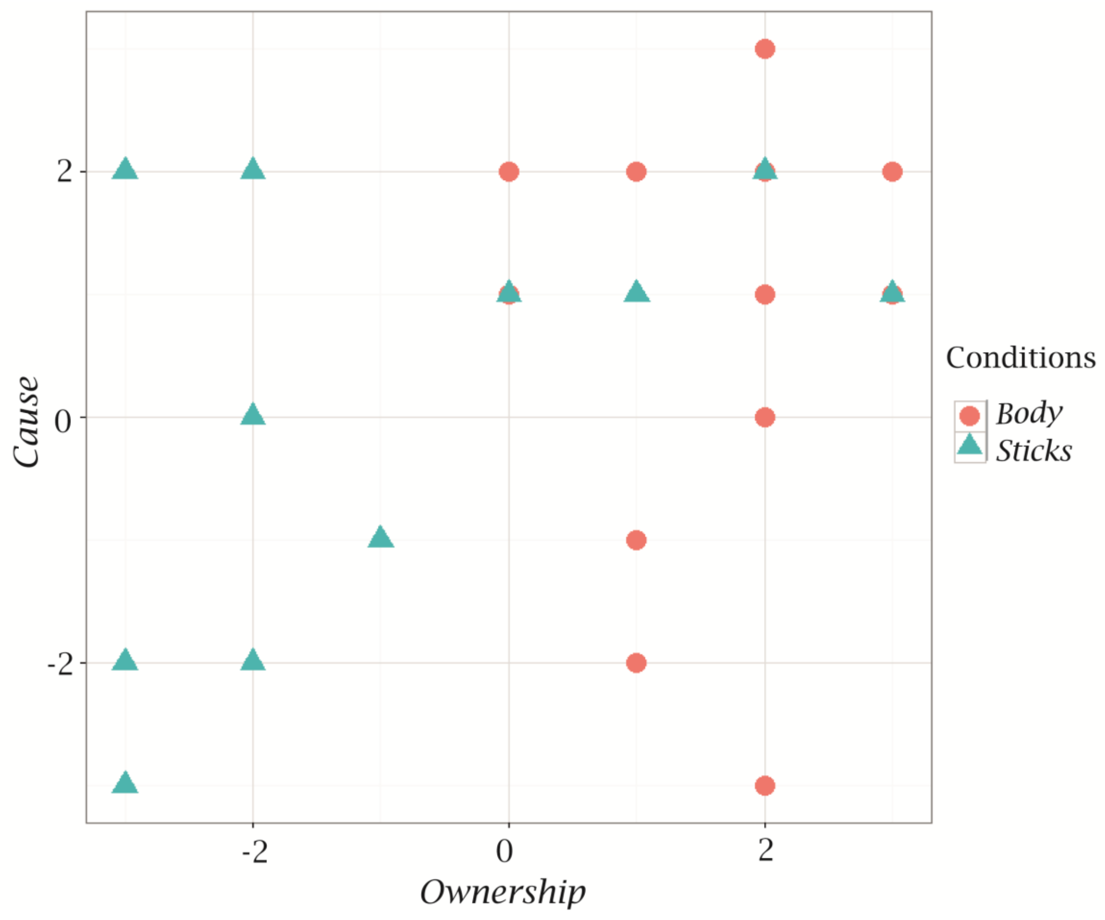

Figure S2. Experiment 2: *ownership* versus *cause* scores. The figure shows the subjective scores rating the feeling of the virtual wheel being the origin of the vibrotactile TOJ cues (*cause* item), and the scores rating the illusory sense of ownership (*ownership* item). Blue triangles and red circles represent scores reported in the *Stick* and *Body* conditions respectively. Scores to the *cause* item have a large variance in both conditions, while scores to the *ownership* item were mostly positive (negative) in the *Body* (*Stick*) condition. Only three subjects out of fourteen reported a positive sense of ownership also in the *Sticks* condition; interestingly the corresponding scores to the *cause* item were also positive

Videos Legends

Video S1. TOJ Visual Cues. The video shows the visual cue adopted in the temporal ordered judgment task, for the different experimental conditions. The cue was a brief (50 ms) rotation of a virtual geared-wheel seen either close but slightly separated from the virtual finger (Exp. 1: No-Touch condition), or in contact with the finger (Exp. 1: Touch condition; Exp. 2: Body condition), or in contact with a wooden stick (Exp. 2: Stick condition). Participants were instructed to keep their gaze fixed on the blue dot depicted on the wheel.

The 3D graphics elements were designed with Autodesk® 3ds Max® and controlled through the Unity® software platform.

Video S2. Visuomotor Task. The video shows the visuomotor task that participants had to perform in between the TOJ blocks. Participants had to move their hands at the position where the green cross was displayed on the virtual table. In Experiment 1 and in the Body condition of Experiment 2, participants' movements were mapped to the virtual body in real time. In the Stick condition of Experiment 2 the virtual sticks were static and participants did not have any visual feedback of their own movements. The 3D graphics elements were designed with Autodesk® 3ds Max® and controlled through the Unity® software platform.
